# Mitochondrial Calcium Deregulation in the Mechanism of Beta-Amyloid and Tau Pathology

**DOI:** 10.3390/cells9092135

**Published:** 2020-09-21

**Authors:** Noemi Esteras, Andrey Y. Abramov

**Affiliations:** Department of Clinical and Movement Neurosciences, UCL Queen Square Institute of Neurology, Queen Square, London WC1N 3BG, UK

**Keywords:** calcium, mitochondria, tau, β-amyloid, MCU, NCLX, VGCCs, glutamate, mPTP

## Abstract

Aggregation and deposition of β-amyloid and/or tau protein are the key neuropathological features in neurodegenerative disorders such as Alzheimer’s disease (AD) and other tauopathies including frontotemporal dementia (FTD). The interaction between oxidative stress, mitochondrial dysfunction and the impairment of calcium ions (Ca^2+^) homeostasis induced by misfolded tau and β-amyloid plays an important role in the progressive neuronal loss occurring in specific areas of the brain. In addition to the control of bioenergetics and ROS production, mitochondria are fine regulators of the cytosolic Ca^2+^ homeostasis that induce vital signalling mechanisms in excitable cells such as neurons. Impairment in the mitochondrial Ca^2+^ uptake through the mitochondrial Ca^2+^ uniporter (MCU) or release through the Na^+^/Ca^2+^ exchanger may lead to mitochondrial Ca^2+^ overload and opening of the permeability transition pore inducing neuronal death. Recent evidence suggests an important role for these mechanisms as the underlying causes for neuronal death in β-amyloid and tau pathology. The present review will focus on the mechanisms that lead to cytosolic and especially mitochondrial Ca^2+^ disturbances occurring in AD and tau-induced FTD, and propose possible therapeutic interventions for these disorders.

## 1. Introduction

Neurodegenerative disorders, characterised by progressive neuronal loss in specific areas of the brain, nowadays represent one of the biggest medical and social challenges: very few therapeutic strategies are available to slow down the course of these diseases. Aggregation and deposition of misfolded proteins are histopathological hallmarks in these conditions. Among them, β-amyloid plaques found in Alzheimer’s disease (AD) and tau aggregates present in AD, frontotemporal dementia (FTD) and up to other 20 diseases collectively termed tauopathies are one of the most studied [1]. Many actors seem to play an essential role in the pathogenesis of these disorders. The interplay between oxidative stress, mitochondrial dysfunction and calcium ions (Ca^2+^) impairment has been shown to mediate neuronal dysfunction and death in patients’ cells and cellular and animal models of β-amyloid and tau pathology. The present review will focus on the Ca^2+^ signalling impairment, with a special emphasis on the mitochondrial Ca^2+^ dysbalance occurring in AD and tau-induced FTD.

## 2. Calcium Homeostasis in Neurons

Ca^2+^ signalling is a key mechanism in critical events for cell life, from gene transcription or cell growth, to cell-specific mechanisms, such as muscle contraction or egg fertilisation. In neurons, Ca^2+^ is involved in most aspects of neuronal function: differentiation and migration, synaptic transmission and plasticity, vesicle release, cell death and survival or neuronal–glial communication [2,3]. Indeed, impairment of Ca^2+^ homeostasis has been widely studied and reported to be crucial in the development of neurodegenerative disorders, such as AD, Parkinson’s disease, amyotrophic lateral sclerosis or Friedrich Ataxia [4,5,6,7].

Ca^2+^ act as second messengers that transmit external signals to its intracellular targets. Ca^2+^ signals are generated by a fine regulation between Ca^2+^ influx and removal, which induces transient fluctuations in the cytosolic [Ca^2+^]. The different kinetics, frequency, amplitudes or spatial locations of these transients entails a signalling mechanism able to exert specific impacts in their downstream effectors [8]. Due to its implications in cellular function, cytosolic Ca^2+^ levels must be therefore tightly regulated, and this becomes essential in excitable cells such as neurons. While a deficient Ca^2+^ signalling might perturb synaptic transmission [9], sustained elevated levels of cytosolic Ca^2+^ are detrimental for neurons: Ca^2+^ overload promotes cell death through different mechanisms, such as necrosis, apoptosis or the more recent ferroptosis, all in which mitochondria play also an essential role [10,11].

**Figure 1:**
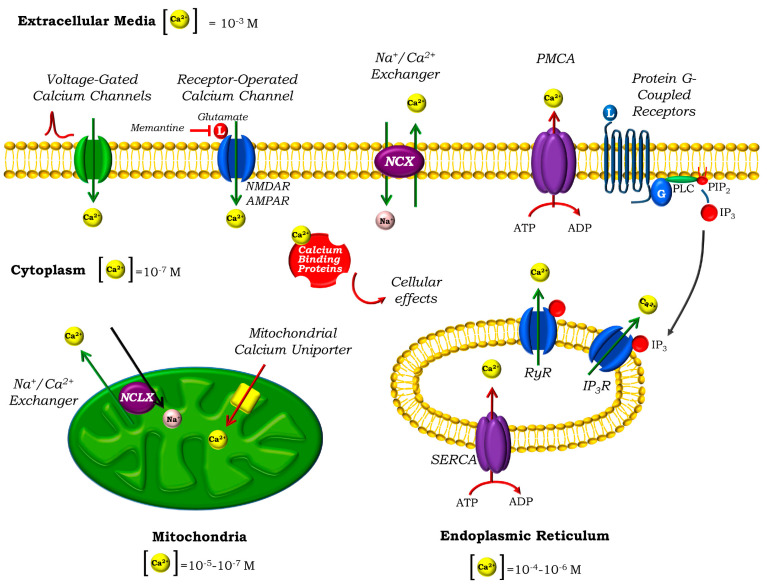
Calcium homeostasis in neurons. Ca^2+^ signals are shaped by a fine regulation between cytosolic Ca^2+^ influx and efflux. The main sources for Ca^2+^ influx are the extracellular media and intracellular stores such as the endoplasmic reticulum (ER). Depolarisation of the neurons leads to the opening of the voltage-gated calcium channels (VGCCs) in the plasma membrane, while ligand binding triggers the opening of the receptor-operated calcium channels (ROCs). AMPA and especially NMDA receptors, both activated by glutamate, are the most important ROCs in the neurons. AD-approved drug memantine is an inhibitor of the NMDARs. Ca^2+^ can also be released to the cytosol from the ER, after activation of the Ryanodine or inositol 1,4,5-triphosphate (IP_3_) receptors. Binding of a ligand (such as glutamate) to a G-protein-coupled receptor in the plasma membrane (such as specific metabotropic glutamate receptors) activates phospholipase C (PLC), leading to the cleavage of the membrane phospholipid phosphatidylinositol 4,5-bisphosphate (PIP_2_), resulting in the release of the soluble second messenger IP3, that diffuses through the cell and binds its receptor. Cytosolic Ca^2+^ binds specific Ca^2+^ binding proteins, which transduce the signal to its final effectors. Excess cytosolic Ca^2+^ is removed from the cytosol by different mechanisms: (i) efflux through the plasma membrane by the Na^+^/Ca^2+^ exchanger NCX and the plasma membrane Ca^2+^-ATPase (PMCA), (ii) uptake to the ER by the sarco/endoplasmic reticulum Ca^2+^-ATPase (SERCA), (iii) uptake to the mitochondria by the mitochondrial Ca^2+^ uniporter MCU and (iv) buffering by Ca^2+^ binding proteins. Mitochondrial Ca^2+^ homeostasis is maintained by the efflux through the mitochondrial Na^+^/Ca^2+^ exchanger NCLX.

In neurons, free cytosolic Ca^2+^ levels are kept at ~100 nM in resting conditions, while the extracellular concentration reaches the millimolar range, defining a substantial concentration gradient of 10^4^. The majority of Ca^2+^ influx from the extracellular site in neurons occurs through different ion channels located in the plasma membrane, either voltage- or ligand-operated, upon specific stimulation (Figure 1). In the first case, depolarisation of the neurons leads to the opening of different voltage-gated Ca^2+^ channels (VGCCs) [12], while in the second, the opening of the ionotropic glutamate receptors triggered by the binding of the excitatory neurotransmitter glutamate is the most important example [13]. Regulated Ca^2+^ release to the cytosol can also occur from intracellular Ca^2+^ stores such as the highly dynamic endoplasmic reticulum (ER) [14]. In this case, agonist binding to Ryanodine (RyRs) or inositol 1,4,5-triphosphate (IP3) receptors leads to a release of Ca^2+^ that plays an important role in many neuronal functions [15]. Depletion of the ER induces the activation of the Store-Operated Calcium Entry (SOCE) in order to replenish the organelle. In neurons, STIM proteins located in the ER sense the decrease in [Ca^2+^], accumulate close to the ER-plasma membrane junctions, and interact with the Store-Operated Calcium Channels (SOCCs) in the plasma membrane, allowing the entrance of Ca^2+^. Orai channels were identified as components of the SOCCs [16], while transient receptor potential channels (TRPC) also play a relevant role [17].

Either way, the duration and spread of the Ca^2+^ signals is controlled by several clearance mechanisms, which dissipate the massive increase in the cytosolic [Ca^2+^] and restore it to its basal levels to maintain Ca^2+^ homeostasis. These mechanisms include the efflux of Ca^2+^ by transporters through the plasma membrane, uptake by organelles such as the ER and the mitochondria and binding to Ca^2+^-buffering proteins.

The main transporters implicated in the efflux of Ca^2+^ out of the neurons are the high-affinity, low capacity plasma membrane Ca^2+^-ATPase (PMCA), which hydrolyses ATP to pump Ca^2+^ against gradient, and the low affinity, high capacity Na^+^/Ca^2+^ exchanger (NCX), which is abundant in neurons and uses the electrochemical gradient of Na^+^ to extrude Ca^2+^ [18]. NCX is reversible, and under specific circumstances of Na^+^ and Ca^2+^ gradient and membrane potential can work in the opposite direction, extruding Na^+^ and letting Ca^2+^ in [19]. The two main intracellular stores that also collaborate in the uptake of cytosolic Ca^2+^ are the mitochondria (which will be discussed in detail later) and the ER, which uses the Sarco-Endoplasmic Reticulum Ca^2+^-ATPase (SERCA) to pump Ca^2+^ into the ER lumen, at the expense of ATP hydrolysis. Finally, several cytosolic Ca^2+^-binding proteins also cooperate in the Ca^2+^ homeostasis by binding and buffering free cytosolic Ca^2+^. The most important belong to the EF-hand family, and include parvalbumin, calbindin D-28k and calretinin, which are expressed in different areas of the brain [20]. Other members of the family, such as calmodulin, S100 proteins or neuronal Ca^2+^ sensors (NCS), are also Ca^2+^-binding proteins that act as Ca^2+^ sensors which transduce the signal to downstream effectors. The latter involve a complex network of signalling cascades that ultimately have specific cellular effects: Ca^2+^-calmodulin kinase II, which regulates long-term potentiation, learning and memory; protein kinase A, which modulates neuronal excitability; or calpains, a family of proteases that cleave amyloid precursor protein APP or tau protein are just a few examples [21,22].

## 3. Mitochondria and Ca^2+^ Homeostasis

Mitochondria play a fundamental role in the rapid buffering and shaping of the cytosolic Ca^2+^ transients. These are mobile organelles, which can be strategically recruited in close proximity to microdomains with a high cytosolic [Ca^2+^], such as the synapses, acting as highly localised Ca^2+^ buffers able to shape the local Ca^2+^ signals and regulate neuronal activity [23].

With additional essential roles, such as ROS production or triggering of apoptosis, the best-known mitochondrial function is controlling the bioenergetics of the cell. Substrate oxidation in the Krebs’ cycle occurs in the matrix and provides the electron transport chain (ETC) in the inner mitochondrial membrane with NADH and FADH_2_. Electrons transfer from these donors to its final acceptor O_2_ through the ETC is coupled with the translocation of protons to the intermembrane space. This creates an electrochemical gradient whose major component is the membrane potential (ΔΨm), which fuels ATP production in the ATP synthase. Importantly, mitochondrial Ca^2+^ uptake activates dehydrogenases at the ETC activating mitochondrial respiration and ATP production [24,25].

In addition to its bioenergetics purpose, ΔΨm is also used by the mitochondria to uptake Ca^2+^ into their matrix through the high capacity, low-affinity mitochondrial Ca^2+^ uniporter (MCU) located in the inner membrane. The molecular composition of the uniporter has been recently elucidated, and involves a protein complex consisting on MCU—the pore-forming component—and several regulatory units (MICU1, MICU2, MCUb, MCUR1 and EMRE) (reviewed in [26]). The role of MICU3, highly expressed in the brain, in enhancing MCU Ca^2+^ uptake has been recently described [27]. Current investigations are focused in understanding the regulation of MCU and the specific role of all these proteins in Ca^2+^ uptake [28,29]. First experiments with MCU knock-out animal models surprisingly revealed that these mice displayed only a mild muscular phenotype [30] and a relatively normal heart function [31]. However, MCU KO in a different genetic background [32] or MICU1 KO appeared to be lethal [33]. Other studies focused in brain function show that silencing of MCU during development induces memory impairment in Drosophila [34], and experiments in brain MCU-KO mitochondria revealed that MCU deletion did not completely block mitochondrial Ca^2+^ uptake, suggesting additional uptake pathways [35]. Indeed, it is still a matter of debate if the complex regulation of MCU can result in alternative uptake modes or if other MCU-independent mechanisms coexist and mediate Ca^2+^ uptake in the mitochondria [36,37].

The mitochondrial Na^+^/Ca^2+^ exchanger NCLX, located in the inner mitochondrial membrane [38], was also molecularly identified not long ago [39] as being responsible for mitochondrial Ca^2+^ efflux in excitable cells. NCLX is a low affinity, high capacity transporter that uses the electrochemical gradient of Na^+^ to extrude Ca^2+^ from mitochondria. Like other Na^+^/Ca^2+^ exchangers, it is related to the plasma membrane NCX, but in addition to Na^+^ is able to exchange Li^+^ for Ca^2+^. Considering that the rate of Ca^2+^ efflux is much slower than MCU-mediated influx, NCLX appears to mediate the rate limiting step in mitochondrial Ca^2+^ homeostasis [40]. Indeed, in contrast to MCU, NCLX deletion is associated with more severe phenotypes in vivo: conditional cardiac NCLX deletion in mice leads to myocardial dysfunction and fulminant heart failure [41]. Inhibition of NCLX in Parkinson’s disease related mutation PINK1 cells leads to mitochondrial Ca^2+^ overload and cell death [42,43]. Regulation of the exchanger activity can occur via different mechanisms such as [Ca^2+^], via direct and indirect mechanisms like calpain-induced degradation, pH, PKC or PKA, as recently reviewed in [44]. In addition, a role for plasma membrane NCX1-3 in mediating mitochondrial Ca^2+^ efflux in brain cells has also been proposed [45,46,47].

Ca^2+^ uptake into the mitochondrial matrix stimulates mitochondrial bioenergetics. Several matrix dehydrogenaseses and metabolite carriers are activated by Ca^2+^, increasing mitochondrial respiration and ATP production [48,49]. This suggests a physiological role for mitochondrial Ca^2+^ in the adaptation of the cell to the energy demands imposed by Ca^2+^ signalling. However, as with cytosolic [Ca^2+^], mitochondrial Ca^2+^ content must be tightly regulated. Mitochondrial Ca^2+^ overload, especially under conditions of oxidative stress, triggers the opening of the mitochondrial permeability transition pore (mPTP), a high-conductance mitochondrial channel whose composition and structure are still under debate. While firmly closed under physiological conditions, after mPTP opening, the mitochondrial inner membrane becomes unselectively permeable to small solutes, leading to ΔΨm collapse and eventually mitochondrial swelling and necrotic and apoptotic cell death. mPTP opening has been implicated as the mechanism of cell death in many human diseases, thus representing a major therapeutic target [50]. It should be noted that ROS are one of the most important triggers for mPTP in combination with Ca^2+^ overload [51]. Misfolded proteins, including β-amyloid and tau, are able to induce ROS production in enzymes (including ETC of mitochondria and NADPH oxidase) or produce free radicals in combination with heavy metals, and trigger mPTP opening [52,53].

## 4. Calcium Homeostasis Impairment in AD and Tauopathies

As mentioned before, Ca^2+^ homeostasis impairment has been linked to many different diseases, including neurodegenerative conditions.

AD is the most common neurodegenerative disorder and the principal cause of dementia. It affects millions of people worldwide, with numbers expecting to multiply in the next years, conveying a critical social and medical challenge. Clinically, AD is characterised by progressive memory loss and cognitive and behavioural impairment [54]. Neuronal and synaptic loss in specific areas of the hippocampus and neocortex, together with the presence of extracellular β-amyloid plaques and intracellular neurofibrillary tangles (NFTs) containing tau aggregates comprise the main neuropathological hallmarks of the disease [55]. Together with them, oxidative stress, mitochondrial dysfunction and altered Ca^2+^ homeostasis have emerged as important actors and been long studied in the last decades to try to unravel the interplay of all these factors in the pathogenesis of the disease [4,56,57]. Although the majority of the cases are sporadic, a small percentage of them are rare familiar cases with an early onset, linked to mutations in the amyloid precursor protein *APP* gene and presenilins 1 and 2 *PSEN1* and *PSEN2* genes, components of the γ-secretase complex involved in the amyloidogenic cleavage of APP that leads to β-amyloid formation [58]. This evidence suggests a critical role for β-amyloid in AD pathogenesis. Indeed, the Amyloid cascade hypothesis, formulated in the early 1990s by Hardy and Higgins [59], proposes that the deposition of β-amyloid is the causative agent of AD pathology, leading to NFTs, neuronal loss and dementia. However, β-amyloid deposits correlate weakly with neuronal death, while spreading of tau pathology through the brain and the number of NFTs are strongly associated with the progression of AD [60]. Tau is a soluble protein that plays a critical role in the stabilisation of the microtubules, but under pathological circumstances self-aggregates into paired-helical fragments (PHF) whose aggregation finally leads to NFTs. Importantly, abnormal tau hyperphosphorylation impacts its pathogenic role and aggregation capacity and indeed deposited tau is highly phosphorylated. In addition, tau isoform imbalance is sufficient to cause neurodegeneration [61]. Interestingly, mutations in the *MAPT* gene encoding tau protein are not linked to AD, but to frontotemporal dementia (FTD) and other tauopathies, a term that comprehends a wider range of neurodegenerative disorders in which deposits of tau are found in the brain [1]. FTD is characterised by the progressive neurodegeneration of frontal and temporal lobes of the brain, and comprises different molecular and clinical entities affecting behaviour, function and language of the patients, which are usually younger than those with AD [62,63]. Research in FTD has gained increasing attention in the recent years, but as in AD, there is still a lot to learn to be able to prevent or cure these disorders.

The important role of Ca^2+^ dysfunction in AD was first proposed by Khachaturian 25 years ago [64]. Growing body of evidence has been published since then, highlighting the multiple molecular mechanisms that can contribute to the Ca^2+^ homeostasis impairment in AD. The role of tau, and especially β-amyloid, has been extensively studied in different animal and cellular models.

### 4.1. Cytosolic Ca^2+^ Disturbances in AD and Tauopathies

β-amyloid was first shown to form Ca^2+^-permeable pores in artificial membranes [65] that lead to dysregulated Ca^2+^ entry in the cytoplasm of brain cells [66,67]. Although less studied, we, and others, have shown that tau is also able to form ion channels under specific conditions [68,69]. Importantly, structure and aggregation stage determined the ability of both proteins to form pores.

Alteration of the glutamatergic signalling, involved in synaptic plasticity, learning and memory, also plays an important role in the Ca^2+^ imbalance and synaptic dysfunction in AD [70,71]. Glutamate is the major excitatory neurotransmitter in the brain and activates a family of metabotropic (G-coupled proteins) and ionotropic (ion channels) receptors. Among the latter, AMPA, and especially NMDA receptors, have attracted much attention due to its role in mediating glutamate excitotoxicity. Excitotoxicity is defined as the neuronal death induced by cellular overload of Ca^2+^ due to excessive stimulation of the glutamate receptors, caused, for example, by an excess of extracellular glutamate. It is involved in the mechanism of cell death in acute (stroke) and chronic neurodegenerative disorders and such has attracted great attention in the pathogenesis of AD [72]. Research has shown that β-amyloid oligomers can directly activate NMDA receptors [73], and specifically those containing the NR2B subunit [74,75]. Receptors expressing this subunit are preferentially localised in the extrasynaptic area and mediate excitotoxicity [76]. It was proposed that modulating the balance between synaptic NR2A and extrasynaptic NR2B may improve behaviour ability in β-amyloid treated mice [77]. Tau involvement in excitotoxicity has been also described in AD [78,79,80] and FTD [81]. For a review of the role of glutamate receptors in AD, see in [82]. Importantly, the non-competitive NMDA receptor antagonist memantine is one of the few drugs approved for use in AD.

The rest of the approved drugs for AD are cholinesterase inhibitors that aim to prevent acetylcholine degradation. Indeed, the cholinergic pathway has been long implicated in AD pathogenesis, and it was proposed that the loss of cholinergic neurotransmission leads to cognitive impairment [83]. Importantly, tau has been shown to play a role in the loss of cholinergic neurons through interaction with muscarinic receptors [84], and some of the toxic effects of β-amyloid are mediated by its interaction with nicotinic acetylcholine receptors. Interestingly, acetylcholine and antibodies against acetylcholine receptors protect neurons against β-amyloid-induced cell death but have no effect on the β-amyloid-induced Ca^2+^ deregulation [85].

Tau and β-amyloid-induced Ca^2+^ dysfunction through VGCCs have been described in different models of AD and tau-induced FTD [86,87]. We have recently shown that in vitro aggregated tau fibrils with the P301S mutation linked to FTD are able to incorporate into membranes and modify their ionic currents, as seen by BLM experiments [69]. When applied to primary neuronal cultures, this leads to the opening of neuronal VGCCs, inducing characteristic Ca^2+^ transients in these cells. Increased cytosolic [Ca^2+^] is able to activate NADPH oxidase, enhancing ROS production in neurons and leading to cell death. Ca^2+^ signals and increased ROS production were observed after the acute application of tau aggregates, suggesting a mechanism by which extracellular tau fibrils can incorporate into the membranes and lead to neuronal dysfunction in the neighbouring neurons [69]. Importantly, we show that tau-induced Ca^2+^ transients and NADPH-driven ROS production were prevented by nifedipine and verapamil, Ca^2+^ channels blockers commonly used in clinic for hypertension. Clinical trials with these compounds in patients with dementia show heterogeneous results, with many demonstrating no positive effect for Ca^2+^ blockers in reducing the rate of cognitive decline in AD patients [88]. However, the severity of the disease at the beginning of the treatment seems to influence the outcome [89]. Indeed, several studies have shown that hypertense patients on treatment with this group of drugs could have a reduced risk of dementia [90,91,92], suggesting a potential use of these medications for the prevention of the disease that needs to be further confirmed.

ER Ca^2+^ dysregulation also plays a role in AD. Many authors have shown an increased Ca^2+^ release from the ER both through RyRs [93,94] and IP3Rs [95,96] by different mechanisms. In addition, impairment of the STIM-mediated SOCE has been described in familiar models of the disease [97,98] and recent studies point at the role of tau in ER stress through TRPC and SOCE upregulation [99].

Ca^2+^ efflux through the plasma membrane by PMCA can be inhibited by β-amyloid and tau, as shown by Mata et al., whose findings are summarised in their review [100]. Both proteins appear to bind the transporter, with tau inhibitory effect occurring in the nanomolar range. In addition, the possible oxidation of PMCA induced by β-amyloid and tau might lead to a decrease in the ATPase activity [101]. β-amyloid is also able to interact with the plasma membrane NCX and reduce its activity [102]. Differing results have been published regarding the protein levels of NCX isotypes in the different areas of brains of patients [103]. However, studies in AD brains and neuronal cultures pointed the specific altered cleavage of NCX3 (and not NCX1) mediated by the Ca^2+^-dependent protease calpain [104]. Interestingly, this feature appeared only in AD brains and not in brains from tauopathies like FTD, suggesting a specific role for β-amyloid and not tau. In addition, both NCX and PMCA can be downregulated in response to oxidative stress [105].

Calpain overactivation has been consistently reported in AD and tauopathy brains [106], and β-amyloid [107] and tauopathy models [108,109]. Other Ca^2+^-dependent molecules, such as calmodulin and its binding proteins have a prominent role in AD [110] and have been suggested as potential biomarkers of the disease [111].

### 4.2. Mitochondrial Ca^2+^ Disturbances in AD and Tauopathies

Neurons, as excitable cells, are continuously firing action potentials and employing a vast Ca^2+^ signalling that comes at the expense of an increased metabolic demand to maintain Ca^2+^ homeostasis (for example through Ca^2+^-ATPases) and re-establish electrochemical gradients. In this context, mitochondria play a fundamental role in maintaining the metabolic needs. This could represent a challenge in neurodegenerative disorders, in which mitochondrial dysfunction has been extensively described together with oxidative stress and Ca^2+^ impairment, all of which are implicated in the pathogenesis of the disease [53,57,112].

In addition, mitochondria themselves are direct sites of action of β-amyloid and tau. β-amyloid has been shown to be imported via TOM [113] and directly produced in this organelle [114], while a fraction of intracellular tau has been found to locate within the inner mitochondrial space [115]. Indeed, mitochondrial accumulation of tau in synaptosomes from AD brains appeared to correlate with synaptic loss [116].

Tau and beta-amyloid dysfunction have been widely linked to altered cytosolic Ca^2+^ homeostasis through the different mechanisms explained before. These scenarios compromise mitochondria in two ways: challenging mitochondrial Ca^2+^ buffering capacity, which might become overloaded, and, in addition, the cellular bioenergetics of cells in which mitochondrial function could be already impaired. Mitochondrial Ca^2+^ uptake by MCU is driven by the ΔΨm, and therefore mitochondrial depolarisation might compromise the uptake of Ca^2+^ and its physiological role in bioenergetics, and in addition expose the cytosol to higher [Ca^2+^]. On the other hand, mitochondrial depolarisation and bioenergetics dysfunction can be triggered by Ca^2+^ and prevented by the inhibition of mitochondrial Ca^2+^ uptake as previously shown in works by Abramov and Duchen [117,118,119].

Several reports highlight the role of the mitochondrial Ca^2+^ uptake in neuronal death induced by glutamate excitotoxicity [120,121]. Qiu et al. showed that MCU overexpression exacerbated excitotoxic cell death, while MCU silencing prevented NMDA-induced mitochondrial Ca^2+^ uptake protecting neurons from excitotoxic cell death [122]. Our group has recently described the protective role of a novel compound, TG-2112x, which is able to partially inhibit mitochondrial Ca^2+^ uptake without affecting ΔΨm or bioenergetics, and protects neurons against glutamate excitotoxicity [123]. These results from Angelova et al. suggest this compound as a new therapeutic opportunity in diseases such as AD in which excitotoxicity play a detrimental role.

Other authors have proposed that the induction of a mild mitochondrial uncoupling with different agents such as non-steroidal anti-inflammatory drugs (NSAIDs) could also reduce mitochondrial Ca^2+^ uptake and prevent overload induced by β-amyloid, protecting neurons against cell death in AD [124]. Results from trials have been however conflicting, probably due to the narrow effective dose window for this strategy, as high doses might lead to opposite effects and collapse the ΔΨm [125].

Recent in vivo imaging by Calvo-Rodriguez et al. in the APP/PS1 transgenic mouse model of AD has shown β-amyloid dependent mitochondrial Ca^2+^ overload in a subset of neurons in the brain of these mice, which preceded neuronal death and could be prevented by MCU inhibition [126]. Interestingly, neuronal death did not occur in neighbour cells with lower mitochondrial Ca^2+^ levels highlighting one more time the deleterious effect of mitochondrial Ca^2+^ overload. This work also evaluated available microarray and RNA-Sequencing datasheets to analyse the expression of mitochondrial Ca^2+^-related genes in patients with AD and found that all the genes involved in mitochondrial uptake were downregulated, while *Slc8b1* gene encoding NCLX was significantly upregulated, suggesting a possible compensatory response to prevent mitochondrial Ca^2+^ overload [126]. However, other reports show contradictory results [127].

The mitochondria-associated ER membranes are subcompartments of the ER connected physically and biochemically to the mitochondria allowing the communication between both organelles and the transfer of Ca^2+^ from ER to mitochondria [128]. Many relevant functions for the pathogenesis of AD such as β-amyloid production appear to occur in these regions [129] and a higher degree of apposition between ER and mitochondria has been found in AD cells, brains and mouse models [130,131]. As seen in preselinin 2 (PS2) cellular and animal models of AD, the increased ER-mitochondria interactions enhance Ca^2+^ transfer, which might contribute to mitochondrial Ca^2+^ overload [132,133]. Some authors have shown that presenilins are able to form cation-permeable pores responsible for passive Ca^2+^ leak from the ER [134], thus contributing to the pathogenesis of the disease, although this hypothesis is under debate, with other authors showing opposite results [135].

mPTP opening induced by mitochondrial Ca^2+^ overload is one of the mechanisms of β-amyloid- and tau-induced mitochondrial dysfunction and cell death [6,8,136,137,138]. β-amyloid is able to interact with a key component of the pore, cyclophilin D, and potentiate mitochondrial dysfunction and mPTP formation [139]. Reduction in cyclophilin D expression, on the other hand, protects neurons and improves learning and memory in mouse models of AD [139]. In addition, treatment with the classical blocker of mPTP, cyclosporine A, or removal of polyphosphate, thought to be a component of the pore, are able to prevent β-amyloid-induced mPTP opening and cell death [119,137].

Impairment of mitochondrial Ca^2+^ efflux has not been explored in the pathogenesis of AD until very recently. Jadiya et al. have shown in different mouse and animal models of the disease that AD progression is associated with the loss of NCLX expression and functionality [127]. Importantly, genetic rescue of NCLX expression in neurons completely restored the cognitive decline and the cellular pathology in the AD mice.

Recent work from our group has shown for the first time the tau-induced altered mitochondrial Ca^2+^ efflux through NCLX in neurons [138]. In this study, we show that K18 tau, a fragment of the protein comprising the four repeat (4R) region of the protein, led to cytosolic Ca^2+^ oscillations in primary neurons after 24 h incubation. These oscillations were followed by mitochondrial Ca^2+^ uptake, and induced a gradual increase in basal cytosolic and mitochondrial Ca^2+^, suggesting an impaired Ca^2+^ handling induced by tau [138]. Stimulation of a physiological Ca^2+^ signal with glutamate (in neurons) or ATP (in astrocytes) incubated with tau further evidenced a slower cytosolic and mitochondrial Ca^2+^ efflux in both cell types. Experiments in permeabilised cells confirmed that the impairment was mediated by NCLX, as showed by the altered Na^+^ and Ca^2+^ currents. More importantly, tau-induced NCLX impairment led to a faster mitochondrial depolarisation when exposing the neurons to (pathological) repetitive Ca^2+^ stimulations, suggesting an increased vulnerability to Ca^2+^-induced cell death [138]. iPSC-derived neurons from patients carrying the FTD-related 10+16 mutation in *MAPT* were also more vulnerable to physiological and pathological Ca^2+^ stimulation, and presented an increased susceptibility to mPTP opening [138]. 

**Figure 2:**
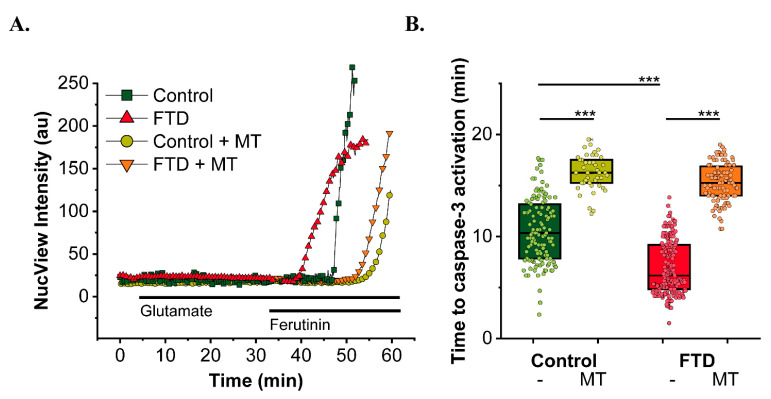
Mitochondrial antioxidants reduce FTD neurons vulnerability to mPTP opening. (**A**) Representative traces depict NucView intensity in iPSC-derived neurons from controls or FTD-related mutation 10+16 in *MAPT* treated or not with MitoTEMPO (MT) 100 nM and exposed to 50 μm glutamate or the electrogenic Ca^2+^ ionophore ferutinin [144,145]. Sudden increase in NucView fluorescence indicates caspase-3 activation. (**B**) Time to caspase-3 activation after Ca^2+^ overload with ferutinin. Box represents median and 25, 75 percentiles. *n* = 126 neurons analysed in control, FTD *n* = 199, control + MT, *n* = 43, FTD + MT, *n* = 104. *** *p* < 0.001, Mann–Whitney test. Method: iPSC-derived neurons were loaded with the non-fluorescent caspase-3 substrate NucView488 for 15 min. NucView is cleaved upon caspase-3 activation inducing a sudden increase in green fluorescence. Images were taken on a Zeiss 710 LSM confocal microscope with an integrated META detection system.

10+16 *MAPT* mutation also impairs neuronal excitability [140] and bioenergetics of iPSC-derived neurons [141]. In contrast to other neurodegeneration models [142,143], 10+16 *MAPT* neurons display an increased mitochondrial membrane potential [141].As a result, mitochondrial ROS production in the neurons is enhanced, leading to oxidative stress and neuronal death, all of which are prevented treating the cells with mitochondrial antioxidants.Oxidative stress, in combination with mitochondrial Ca^2+^ overload, are the triggers for mPTP opening. Preliminary data shows that mitochondrial antioxidants are able to protect the 10+16 neurons and reduce their susceptibility to mPTP opening (Figure 2). Confirming previous results [138], cellular Ca^2+^ overload induced by the ionophore ferutinin triggered mPTP opening and led to apoptosis in iPSC-neurons, which occurred significantly earlier in the FTD patients than in controls (Figure 2). Treatment with the mitochondrial antioxidant MitoTEMPO (MT, 1 h, 100 nM) significantly delayed the mPTP opening in the patients’ neurons to times similar to control, thus counteracting their increased vulnerability. This, together with previous results [141], highlights the potential role of mitochondrial antioxidants in the prevention of neuronal death through different mechanisms that might include averting mitochondrial Ca^2+^ overload. Further investigations will be needed to prove this point and understand if this effect is merely due to the reduction of the already elevated mitochondrial ROS, or if mito ROS overproduction induced by tau might influence other aspects of cytosolic or mitochondrial Ca^2+^ homeostasis trough different mechanisms such as redox regulation. These results highlight the close interconnection between impaired bioenergetics, oxidative stress and Ca^2+^ signalling in tau pathology.

## 5. Conclusions

Ca^2+^, and especially mitochondrial Ca^2+^ homeostasis, plays a key role in neurodegenerative disorders including AD and other tauopathies like FTD. As detailed in the present review, both β-amyloid and tau protein induce cytosolic and mitochondrial Ca^2+^ deregulation through different direct and indirect pathways that ultimately lead to neuronal dysfuntion and cell death. Importantly, isoform type, length, or aggregation stage, among other characteristics of these proteins have been shown to influence the pathogenic mechanism. Mitochondrial Ca^2+^ overload appears as a downstream key event in the process of neurodegeneration, and recent studies point at a direct role of these proteins in the impairment of mitochondrial Ca^2+^ influx (β-amyloid) and efflux (β-amyloid and tau). Specific targeting of the mechanisms leading to Ca^2+^ impairment, and especially mitochondria-targeted therapies emerge as potential treatments for these disorders.
